# Endocrine and cardiovascular consequences of plasticiser exposure: a narrative review from the thyroid- cardiac axis perspective

**DOI:** 10.3389/fphar.2026.1876287

**Published:** 2026-08-28

**Authors:** Debarati Halder, Varshini P.V, Tridip Mitra, Divyapriya Karthikeyan, A. H. Sruthi Anil Kumar, Meenakshi Sundari S.N, Snehasis Mishra, Rajiv Janardhanan

**Affiliations:** 1 Division of Medical Research, Faculty of Medicine and Health Sciences, SRM Institute of Science and Technology, Kattankulathur, Tamil Nadu, India; 2 Department of General Medicine, Faculty of Medicine and Health Sciences, SRM Institute of Science and Technology, Kattankulathur, Tamil Nadu, India

**Keywords:** cardiovascular diseases, hypothalamic-pituitary- thyroid axis, hypothyroidism, microfluidics, plasticisers

## Abstract

Endocrine-disrupting chemicals particularly plasticisers such as phthalates, bisphenols and their emerging substitutes, are increasingly recognised as the main environmental toxicants with significant implications for thyroid and cardiovascular health. Due to their widespread use in consumer products, food packaging, medical devices, cosmetics and industrial materials, chronic human exposure occurs *via* ingestion, inhalation, dermal absorption and parenteral administration. Increasing evidence indicates that these compounds disrupt the hypothalamic-pituitary-thyroid axis by interfering with thyroid hormone synthesis, transport, receptor signalling, iodine homeostasis and endocrine feedback regulation. These disruptions contribute to thyroid autoimmunity, hypothyroidism, and broader endocrine imbalance. Because tfundihyroid hormones are vital for cardiovascular health, plasticiser-related thyroid problems can indirectly lead to cardiovascular issues, including hypertension, endothelial dysfunction, dyslipidaemia, arrhythmias, atherosclerosis and heart failure. Mechanistically, these effects are mediated by oxidative stress, mitochondrial dysfunction, inflammatory signalling, ion-channel disruption, metabolic dysregulation and epigenetic modifications. Integrated thyroid-cardiac crosstalk is increasingly recognised as a critical mechanistic axis linking environmental EDC exposure to the progression of cardiometabolic disease. The review highlights the emerging biomarkers and advanced microfluidic and organ-on-chip technologies as promising tools for early detection and mechanistic evaluation of plasticiser-induced thyroid-cardiac dysfunction. Collectively, this review also highlights the critical need for long-term human studies, combined exposure assessments and translational diagnostic approaches to enhance our understanding and alleviate the escalating public health burden of environmental plasticiser exposure.

## Graphical Abstract

**Figure:**
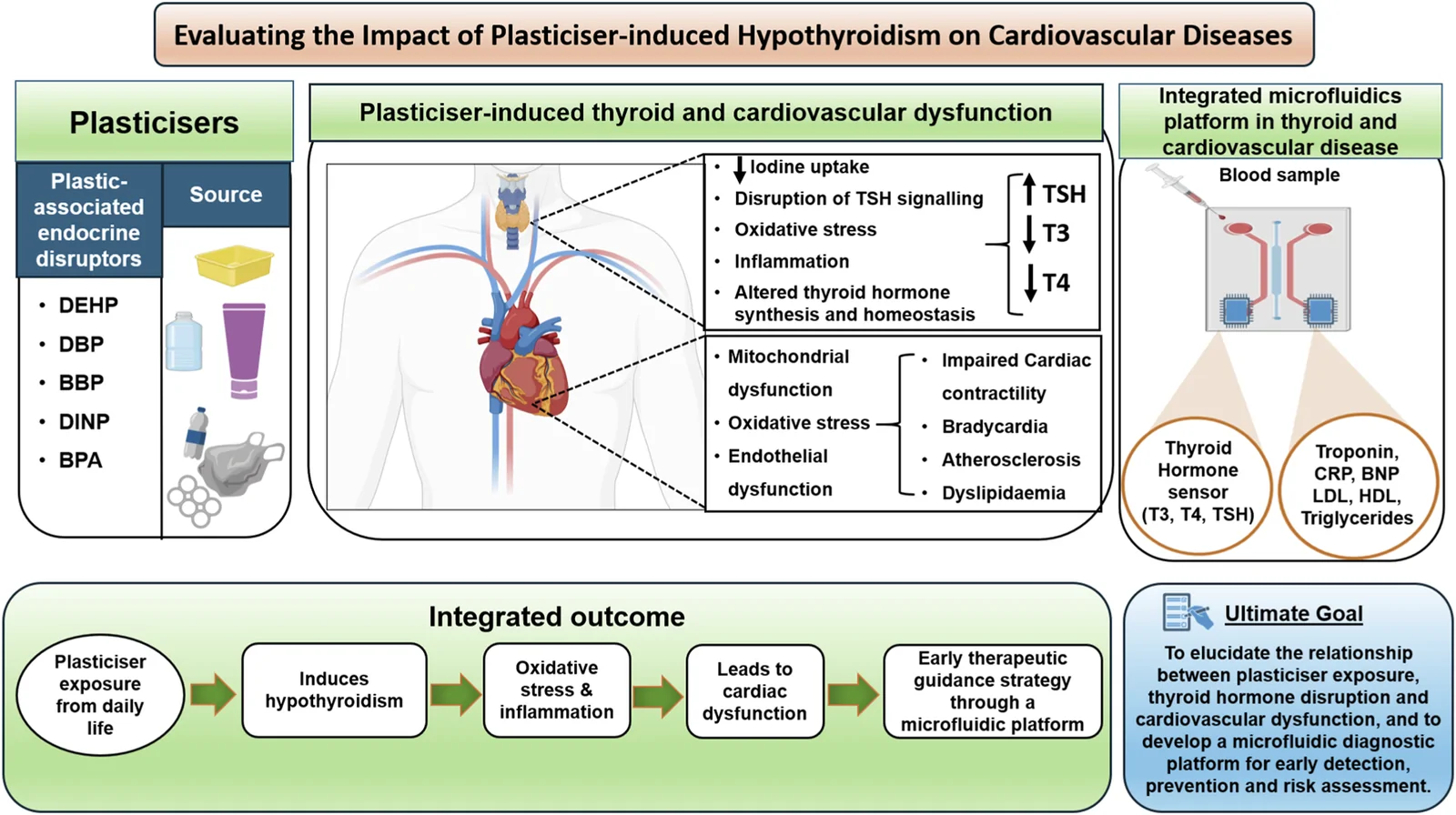
Plasticiser induced thyroid and cardiac dysfunction and early diagnosis by a microfluidic platform. Schematic representation illustrates that plasticiser disrupts thyroid hormone regulation, causing oxidative stress, inflammation and cardiac abnormalities and a microfluidic platform integrated for early diagnosis. Common plasticisers such as DEHP, DBP, BBP, DINP and BPA, which originate from everyday consumer products, may interfere with thyroid function by reducing iodine uptake, disrupting TSH signalling and inducing oxidative stress and inflammation. These alterations lead to changes in thyroid hormone levels (TSH, T3 and T4), contributing to mitochondrial dysfunction, endothelial damage and oxidative stress in cardiac tissues. These contribute to cardiac contractility, bradycardia and dyslipidaemia. Using a microfluidic platform, early thyroid hormone markers and cardiovascular markers can be identified early. These platforms can be used for better prevention and diagnostic strategies.

## 1 Introduction

Endocrine-disrupting chemicals (EDCs) are natural or synthetic substances that interfere with hormonal signalling by mimicking, blocking, or altering endocrine function, leading to adverse health outcomes (Guarnotta et al., 2022). Commonly found in plastics, pesticides, personal care products and food packaging, their widespread presence results in continuous human exposure through dietary, behavioural and residential pathways (Mitra et al., 2024). Among these, plasticisers, are the most prevalent EDCs encountered in daily life (Dalamaga et al., 2024). Plasticisers disrupt thyroid endocrine function through multiple molecular mechanisms that are discussed in later sections (Sikka and Wang, 2008; Chen et al., 2022). Among the endocrine systems affected, disruption of the hypothalamic-pituitary-thyroid (HPT) axis (Sousa-Vidal et al., 2022) has emerged as one of the principal mechanisms linking plasticiser exposure with cardiovascular disease (CVD) (Xega et al., 2026).

The HPT axis is a well-orchestrated neuroendocrine regulatory system that maintains systemic thyroid hormone homeostasis (Levie et al., 2018). Disruption of this axis by plasticisers impairs endocrine regulation, promotes inflammatory signalling, and contributes to metabolic imbalance (Levie et al., 2018), thereby increasing susceptibility to thyroid dysfunction and subsequent cardiovascular disease (Bereketoglu and Pradhan, 2022; Mohammadi and Ashari, 2021). Consequently, thyroid dysfunction may contribute to CVD through mechanisms discussed later in this review (Cappola et al., 2019; Biondi and Cappola, 2022).

Epidemiological evidence indicates a substantial burden of CVD among individuals with thyroid dysfunction (Cappola et al., 2019). Subclinical hypothyroidism affects approximately 5%–10% of the population and is associated with increased risks of coronary heart disease, heart failure (HF) and stroke, particularly among young- and middle-aged individuals (Biondi and Cappola, 2022). Hyperthyroidism has an estimated prevalence of 0.5%–1.5% and is associated with an increased risk of ischaemic heart disease and stroke, with hazard ratios ranging from 1.11 to 1.35 for major cardiovascular events. It has also been reported in approximately 0.3%–0.7% of patients presenting with myocardial infarction (Hussein et al., 2021). Furthermore, a meta-analysis encompassing 55 studies and 1,898,314 participants reported that overt hypothyroidism was associated with increased incidence of myocardial ischemia (13%), myocardial infarction (15%) and arrhythmias (96%), compared with euthyroid individuals (Corona et al., 2021). Meta-analyses further demonstrate that low triiodothyronine (T3) syndrome and hypothyroidism are associated with increased all-cause mortality and adverse cardiovascular outcomes, particularly among patients with acute coronary syndrome (Chang et al., 2020; Aziz et al., 2017; Yamakawa et al., 2021).

The present review critically synthesises current molecular experimental and epidemiological evidence linking plasticiser exposure with thyroid dysfunction and CVD. Particular emphasis is placed on the mechanisms underlying HPT-axis disruption, thyroid-cardiac crosstalk, and the role of oxidative stress, mitochondrial dysfunction, inflammation, ion-channel dysregulation and epigenetic alterations in cardiovascular pathology. The review further discusses emerging biomarkers, advances in microfluidic and organ-on-chip technologies, and existing translational gaps to provide a comprehensive framework for understanding plasticiser-induced thyroid-cardiac dysfunction (Graphical Abstract).

## 2 Review of literature

A structured narrative review methodology was adopted to synthesise current evidence on the relationship between plasticiser exposure, thyroid dysfunction and cardiovascular disease. A comprehensive literature search was performed using Google Scholar, PubMed, ScienceDirect, Springer and ResearchGate databases. Most common keywords used to find publications were “Thyroid dysfunctions”, “Cardiovascular diseases”, “EDC”, “Plasticisers”, “HPT axis”. The term “AND” was used when searching for two or more keywords at the same time, to ensure no relevant publications were overlooked. The publications were manually reviewed and then filtered based on the title, abstract and content (Table 1).

**TABLE 1:** This table summarizes experimental, epidemiological and narrative review-based studies assessing the effects of EDCs.

Study model	Country	EDC	Exposure	Sample analysed	Quantification method	Major health impact	Ref.
A. Human epidemiological studies							
Pregnant women (SELMA cohort)	Sweden (European)	DEHP, DINP, DBP, BBzP metabolites	Urinary molar sums (higher exposure quartiles)	Maternal urine (early pregnancy)	SPE coupled with LC-MS/MS or HPLC-MS/MS for molar sums (ng/mL range, LOD ∼0.1–1 ng/mL)	Reduced FT4 and TT4, increased TSH/FT4 ratio, associated with preterm birth and impaired fetal growth	19
B. Animal studies							
Rat	NS	PS-NPs	1–10 mg/kg/day for 5 weeks	Serum	Not detailed (hormone assays)	Reduced T3, FT3 and FT4 indicating thyroid dysfunction	32
Mouse	NS	PS-MPs ≤10 μm	Size-dependent exposure	Testicular tissue	Histology and hormone assays	Reduced testosterone, impaired sperm quality, blood-testis barrier disruption and inflammation	
Rat	NS	PAEs	NR	Testicular tissue	Biochemical assays (ACP, SOD, MDA)	Spermatogenic disruption and oxidative stress	
Rat	NS	PS-MPs	Developmental exposure	Ovaries and granulosa cells	Histology, AMH and estradiol assays	Reduced follicular growth and irregular estrous cycle	
Female Wistar rat	China	DBP with iodine supplementation	DBP 4 mg/kg/day for 35 days	Serum	ELISA, immunohistochemistry, ROS assays	Exacerbated autoimmune thyroiditis, oxidative stress and inflammatory responses	33
Rat	NS	PAEs contaminated with microplastics	NR	Testicular tissue	HPLC-MS/MS and enzymatic assays	Oxidative stress, reduced sperm quality and impaired spermatogenesis	19
C. *In Vitro* studies							
No independent *in vitro* studies identified among the cited references. Studies involving isolated granulosa cells were performed as part of animal experiments and are therefore included under animal studies							
D. *In Silico* studies							
Molecular docking	Saudi Arabia	DEHP, DINCH, ATBC, DEHA	Computational	Thyroid hormone signalling proteins	Schrodinger induced Fit docking	Predicted disruption of T3/T4 signalling and HPT axis homeostasis	40

Study type	Country	EDC(s) reviewed	Scope	Main conclusion	Ref.
E. Review studies					
Narrative review	Global	BPA	Human, animal and cell-based studies	BPA promotes endocrine disruption, carcinogenesis and epigenetic alterations	37
Review	Poland	Phthalates, BPA, PCBs, pesticides, heavy metals	Human environmental exposure	Dietary EDC exposure disrupts thyroid hormone synthesis, transport and metabolism	46
Review	Global	PCBs, PBDEs, BPA, phthalates, pesticides, dioxins, organotin	Human and animal studies	EDC exposure is associated with reproductive disorders, thyroid dysfunction, metabolic disease and cancer	48

Abbreviations: NS, not specified; PS-NPs, Polystyrene nano plastics; PS-MPs, Polystyrene microplastics; AMH, Anti-Müllerian Hormone; PAEs, Phthalate esters; HPLC-MS/MS, High-Performance Liquid Chromatography-Tandem Mass Spectrometry; ACP; SOD; MDA, acid phosphatase; Superoxide Dismutase; Malondialdehyde; DEHP, di (2-ethylhexyl) phthalate; DINP, diisononyl phthalate; DBP; dibutyl phthalate; BBzP - benzyl butyl phthalate; DINCH, Diisononyl Cyclohexane-1, 2-dicarboxylate; ATBC, acetyl tributyl citrate; DEHA, diethyl hydroxylamine; BDE, bromo diphenyl ether; TBG, thyroxine-binding globulin; SPE, solid phase extraction; LC-MS/MS, liquid chromatography-tandem mass spectrometry; ELISA, Enzyme-linked immunosorbent assay; ROS, assays, Reactive Oxygen Species; HPT, hypothalamic-pituitary-thyroid; BPA, Bisphenol A; PCB, polychlorinated biphenyls; EDC, Endocrine-Disrupting Chemicals; PBDEs, Polybrominated diphenyl ethers.

## 3 Exposure patterns of plasticisers to humans

Plasticisers are synthetic chemicals widely incorporated into consumer products to improve the flexibility, durability and stability of plastic materials (Plunk and Richards, 2020). Because most plasticisers are not covalently bound to polymer matrices, they readily migrate into the surrounding environment, resulting in continuous human exposure through ingestion, inhalation, dermal absorption and parenteral administration (Derakhshan et al., 2021). Major exposure sources include food and beverage packaging, indoor dust, drinking water, personal care products, household materials and medical devices, making exposure ubiquitous across all age groups (Derakhshan et al., 2021).

### 3.1 Phthalates

Among the numerous plasticisers currently in commercial use, phthalates and bisphenols are the two most extensively studied classes because of their widespread applications and well-established endocrine-disrupting properties (Ramadan et al., 2020). Phthalates are diesters of phthalic acid primarily used to improve the flexibility of polyvinyl chloride (PVC) and other polymeric materials, whereas bisphenols are diphenolic compounds widely employed in the manufacture of polycarbonate plastics, epoxy resins, thermal paper and food-contact materials (Derakhshan et al., 2021; Park et al., 2019).

Human exposure occurs through multiple environmental, occupational and medical pathways (Sforzi et al., 2026). Dietary intake remains the predominant route because plasticisers readily migrate from food packaging, processing equipment and storage containers into foods and beverages, particularly fatty foods, processed meals, canned products, dairy products, seafood and bottled water (Sforzi et al., 2026; Mastanjević et al., 2025). Additional exposure occurs through inhalation and ingestion of indoor air and household dust, dermal absorption from cosmetics and personal care products and parenteral exposure from medical devices (Sforzi et al., 2026; Mastanjević et al., 2025; Parlett et al., 2013; Šimunović et al., 2022). Occupational exposure is especially relevant among workers involved in plastic manufacturing, healthcare, waste recycling, construction, printing and cosmetic industries, where prolonged inhalation and dermal contact may substantially increase body burdens (Parlett et al., 2013).

Phthalates are among the most widely used plasticisers owing to their ability to improve the flexibility, transparency and durability of plastics (Ramadan et al., 2020). Common phthalates include DEHP, DBP, benzyl butyl phthalate (BBP), diisononyl phthalate (DINP) and diisodecyl phthalate (DIDP) (Ramadan et al., 2020). DEHP and DINP are widely used in PVC-based products like intravenous tubing, blood bags, flooring materials, cables and food packaging films whereas DBP and BBP are commonly used in adhesives, printing inks, coatings and cosmetic formulations such as nail polish and fragrances (Giuliani et al., 2020). Consequently, dietary intake, consumer products and medical devices represent the principal sources of phthalate exposure in the general population.

### 3.2 Bisphenols

Bisphenol A (BPA) and its structural analogues, bisphenol S (BPS), bisphenol F (BPF) and bisphenol AF (BPAF), are mainly present in polycarbonate plastics, epoxy resin lining of food cans, thermal receipt papers, reusable water bottles and dental sealants. Although regulatory restrictions have reduced BPA use in several countries, structurally related analogues such as BPS and BPF are increasingly replacing BPA in commercial products (Larsson et al., 2017). However, growing toxicological evidence indicates that many of these substitutes possess endocrine-disrupting properties comparable to conventional bisphenols, raising concerns regarding “regrettable substitution” (Shoaff et al., 2019).

Similarly, restrictions on several conventional phthalates have accelerated the use of alternative non-phthalate plasticisers, including di-isononyl cyclohexane-1,2-dicarboxylate (DINCH), acetyl tributyl citrate (ATBC), di (2-ethylhexyl) adipate (DEHA) and epoxidised soybean oil (ESBO) in medical devices, food-contact materials, children’s toys and packaging products (Shoaff et al., 2019). Although these alternatives were introduced as safer substitutes, emerging toxicological studies suggest that several replacement plasticisers exhibit endocrine-disrupting, thyroid-disrupting and metabolic effects similar to those reported for conventional phthalates and bisphenols (Shoaff et al., 2019).

Exposure profiles vary considerably across different populations. Pregnant women and fetuses (Sforzi et al., 2026) are particularly vulnerable because several plasticisers, including DEHP, DINP, BPA and BPF, as they readily cross the placental barrier and accumulate in amniotic fluid, placental tissue and cord blood, potentially disrupting fetal thyroid hormone homeostasis and metabolic programming (Fisher et al., 2019). Infants and children experience proportionally greater exposure because of immature detoxification systems, increased gastrointestinal absorption, behavioural factors and developmental vulnerability (Fisher et al., 2019). Premature infants receiving intensive medical care constitute one of the highest-exposed populations because repeated use of plastic-containing medical devices can result in substantial systemic exposure during critical developmental windows (Šimunović et al., 2022; Kuiper et al., 2025).

### 3.3 Other metabolites

Biomonitoring studies consistently demonstrate the widespread presence of plasticiser metabolites, including mono-(2-ethylhexyl) phthalate (MEHP), mono-benzyl phthalate (MBzP), BPA-glucuronide and DINCH metabolites in urine, serum, breast milk, placental tissue and even in follicular fluid indicating ongoing and lifelong human exposure (Fisher et al., 2019). Individual susceptibility to plasticiser toxicity is influenced by sex, developmental stage, iodine status, genetic background and cumulative exposure burden (Fisher et al., 2019). Moreover, simultaneous exposure to multiple plasticisers may produce additive, synergistic, or antagonistic biological effects through convergent molecular pathways involving thyroid hormone receptors (TRs), estrogen receptors (ERs), peroxisome proliferator-activated receptors (PPARs), oxidative stress pathways and epigenetic regulation (Ghisari and Bonefeld-Jorgensen, 2009). These complexities highlight the challenges in accurately assessing the health risks of plasticiser exposure and underscore the need for integrated mechanistic, epidemiological and translational studies define the contribution of plasticiser exposure to thyroid dysfunction, metabolic syndrome and CVD.

## 4 Mechanisms of thyroid dysfunction and impact of plasticisers

### 4.1 Thyroid hormone disruption by plasticisers

#### 4.1.1 Phthalates

Plasticisers disrupt thyroid homeostasis by impairing hormone synthesis, iodine uptake, hormone transport, receptor signalling and endocrine feedback regulation, ultimately impairing thyroid hormone homeostasis and HPT-axis function (Ramadan et al., 2020; Ullah et al., 2023).

Phthalates are among the most studied plasticisers (Derakhshan et al., 2021). Epidemiological studies have associated urinary phthalate metabolites with altered thyroid hormone concentrations, thyroid autoimmunity, autoimmune thyroiditis and changes in anti-thyroid peroxidase (anti-TPO) antibody levels, indicating that chronic exposure may contribute to endocrine dysfunction (Bereketoglu and Pradhan, 2022). These associations are particularly important during pregnancy, where maternal thyroid hormone disruption may impair fetal neurodevelopment and endocrine programming. Evidence also suggests that the sex-specific susceptibility, likely reflecting differences in hormonal milieu, receptor expression, developmental stage and xenobiotic metabolism (Plunk and Richards, 2020).

*In vivo* studies have shown that exposure to DEHP, DBP and related congeners alters the expression of genes involved in thyroid hormone synthesis and signalling, including sodium/iodide symporter (NIS) (Duan et al., 2018), TPO (Bereketoglu and Pradhan, 2022), transthyretin (TTR) (Liu et al., 2015) and TRs (Jia et al., 2016). Chronic exposure further induces long-term transcriptional reprogramming and histopathological alterations within the thyroid gland, supporting persistent disruption of thyroid homeostasis (Jia et al., 2016). Complementar*y in vitro* studies confirm impaired thyroid hormone receptor signalling and reduced cellular responsiveness to thyroid hormone responsiveness following phthalate exposure (Bereketoglu and Pradhan, 2022).

#### 4.1.2 Bisphenols

Bisphenol and its analogues exhibit broader receptor-mediated thyroid-disruptive activities than many phthalates (Faria et al., 2019). Unlike phthalates, they function as ER agonists, while antagonising thyroid hormone receptor signalling. They also activate oncogenic signalling pathways such as c-MYC, inhibit tumour suppressor proteins such as Phosphatase and tensin homolog deleted on chromosome 10 (PTEN) and promote oxidative stress, collectively contributing to thyroid dysfunction (Faria et al., 2019). Several bisphenol analogues exhibit distinct receptor-binding profiles, highlighting important differences in their endocrine-disrupting potential (Khan et al., 2021).

Epidemiological studies have associated bisphenol exposure with thyroid dysfunction, including altered thyroid hormone concentrations, thyroid hyperplasia and nodular goitre, particularly under conditions of excessive iodine intake (Hu et al., 2023). These findings suggest that chronic exposure to bisphenols may contribute to thyroid pathology through multiple endocrine-disrupting mechanisms. *In vivo* studies further support these observations by demonstrating thyroid-disruptive effects following bisphenol exposure, including alterations in thyroid morphology and endocrine function (Khan et al., 2021). They have shown that bisphenol analogues differ in their receptor-binding properties. BPA exhibits pronounced antagonistic activity towards TRβ, whereas BPAF demonstrates greater estrogenic potency and receptor-binding affinity, indicating that relatively small structural modifications among bisphenol analogues can substantially influence their endocrine-disrupting potential (Khan et al., 2021; David et al., 2015).

#### 4.1.3 Alternative plasticisers

Emerging evidence indicates that several alternative plasticisers, including DINCH, ATBC and DEHA, may exhibit thyroid-disrupting effects comparable to conventional phthalates despite being introduced as safer replacements (Shoaff et al., 2019). *In vivo* toxicological studies have reported no significant changes in behaviour, serum chemistry, organ weights, or reproductive outcomes following exposure to DINCH (David et al., 2015). However, prolonged high-dose exposure (300–1,000 mg/kg body weight/day) led to thyroid hyperplasia and histopathological alterations, suggesting thyroid toxicity under certain exposure conditions (David et al., 2015). Molecular docking analyses demonstrate that DINCH, ATBC and DEHA, exhibit strong affinity for TRα, with some compounds demonstrating receptor-binding affinities comparable to or greater than the endogenous ligand triiodothyronine (T3) (Zughaibi et al., 2022). Notably, DINCH and ATBC displayed greater interaction overlap and receptor-binding stability than DEHP, providing mechanistic support for their potential thyroid-disrupting activity (Zughaibi et al., 2022). In contrast, ESBO currently lacks sufficient mechanistic evidence linking it directly to thyroid hormone disruption, highlighting an important knowledge gap regarding the safety of several emerging replacement plasticisers (Zheng et al., 2025).

### 4.2 HPT axis dysregulation by plasticisers

The HPT axis is the principal neuroendocrine system responsible for maintaining thyroid hormone homeostasis through coordinated interactions between hypothalamus, pituitary gland and thyroid gland (Levie et al., 2018). Under physiological conditions, low circulating thyroid hormone stimulates hypothalamic secretion of thyrotropin-releasing hormone (TRH) and the pituitary gland to secrete thyroid-stimulating hormone (TSH), which promotes iodine uptake and thyroid hormone synthesis. Rising concentrations of T3 and T4 subsequently suppress TRH and TSH secretion through negative feedback, maintaining endocrine homeostasis (Levie et al., 2018).

Disruption of thyroid hormone homeostasis by plasticisers ultimately destabilise the HPT axis by impairing normal endocrine feedback regulation between the hypothalamus, pituitary gland and thyroid gland (Zoeller et al., 2005). Declining circulating T3 and T4 concentrations trigger compensatory increases in TSH to restore thyroid hormone production. However, persistent plasticiser exposure overwhelms these adaptive responses, resulting in chronic HPT-axis dysregulation, sustained thyroid stress and progressive thyroid dysfunction (Köhrle and Frädrich, 2021; Rodrigues et al., 2024).

Chronic disruption of the HPT axis extends beyond impaired endocrine regulation and has widespread systemic consequences. Altered thyroid hormone availability modifies gene expression, cellular metabolism and tissue responsiveness, leading to disturbances in multiple physiological processes, including lipid metabolism, vascular homeostasis and mitochondrial energy production (Schug et al., 2011; Sokal et al., 2021; Ghassabian and Trasande, 2018). Prolonged endocrine imbalance may also induce persistent alterations in intracellular signalling pathways and epigenetic regulation, contributing to long-term metabolic and cardiovascular dysfunction (Schug et al., 2011; Sokal et al., 2021).

**FIGURE 1:**
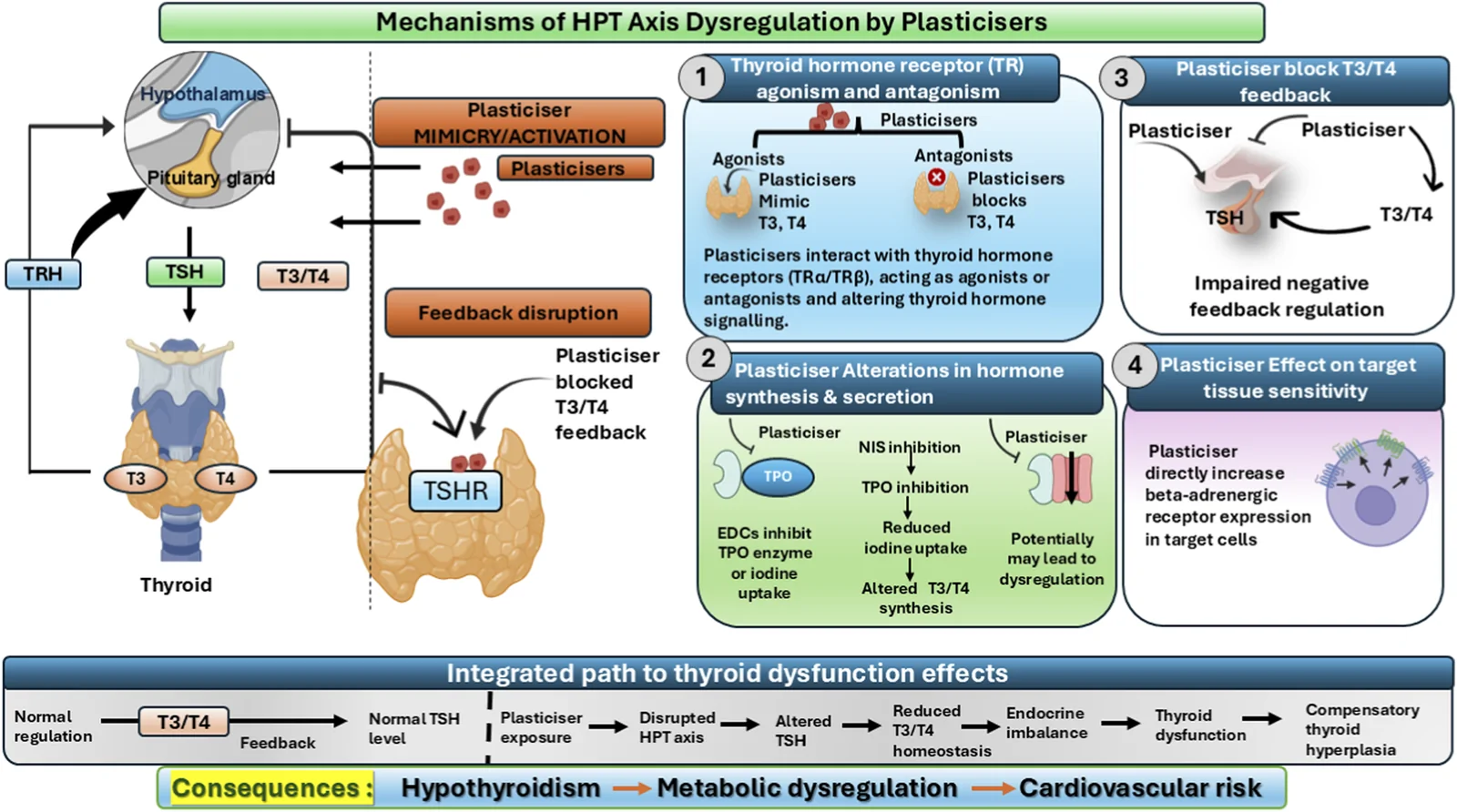
HPT axis disruption and thyroid dysfunction induced by Plasticiser exposure. Plasticisers dysregulate the HPT axis through multiple complementary mechanisms involving thyroid hormone synthesis, receptor signalling, endocrine feedback regulation and peripheral tissue responsiveness, ultimately promoting thyroid dysfunction. Under normal physiological conditions, TRH released from the hypothalamus stimulates pituitary secretion of TSH, which subsequently promotes thyroidal synthesis and secretion of T3 and T4. Circulating T3/T4 maintain endocrine homeostasis through negative feedback regulation of the hypothalamus and pituitary gland. Plasticisers, including phthalates, bisphenols and alternative Plasticisers, disrupt this regulatory network by: (1) mimicking or antagonizing T3/T4 activity through interaction with TRs and TSHR; (2) impairing thyroid hormone synthesis and secretion through inhibition of NIS, TPO and iodine uptake pathways; (3) dysregulating endocrine feedback mechanisms, leading to sustained abnormal TSH signalling and altered T3/T4 homeostasis; and (4) modifying peripheral target tissue sensitivity through altered receptor expression and intracellular signalling pathways. Collectively, these mechanisms contribute to dysregulated thyroid hormone production, amplified systemic thyroid responses, thyroid hyperplasia, endocrine imbalance and increased risk of thyroid dysfunction.

Because thyroid hormones are essential regulators of myocardial contractility, vascular tone, endothelial function, lipid metabolism and mitochondrial bioenergetics, persistent HPT-axis dysregulation has important cardiovascular consequences (Ghassabian and Trasande, 2018; Diamanti-Kandarakis et al., 2009; Klein and Ojamaa, 2001). Plasticiser-induced reductions in T3 and T4 may therefore contribute to dyslipidaemia, endothelial dysfunction, hypertension, atherosclerosis and impaired myocardial performance (Jabbar et al., 2017; Rodondi et al., 2010; Biondi and Cooper, 2008). Epidemiological studies further associate chronic plasticiser exposure with altered thyroid hormone profiles, elevated TSH concentrations and thyroid autoimmunity, supporting hypothyroidism as an important intermediary linking plasticiser exposure with cardiovascular disease (Gore et al., 2015; Boas et al., 2012) (Figure 1).

## 5 Mechanism of cardiac dysfunction and impact of plasticisers

Plasticisers induce cardiovascular toxicity through both direct cellular effects and indirect endocrine mechanisms (Klein and Danzi, 2007). Accordingly, plasticiser-induced cardiovascular disease should be viewed as a multifactorial process involving both endocrine and non-endocrine mechanisms.

### 5.1 Molecular mechanisms and pathways

**FIGURE 2:**
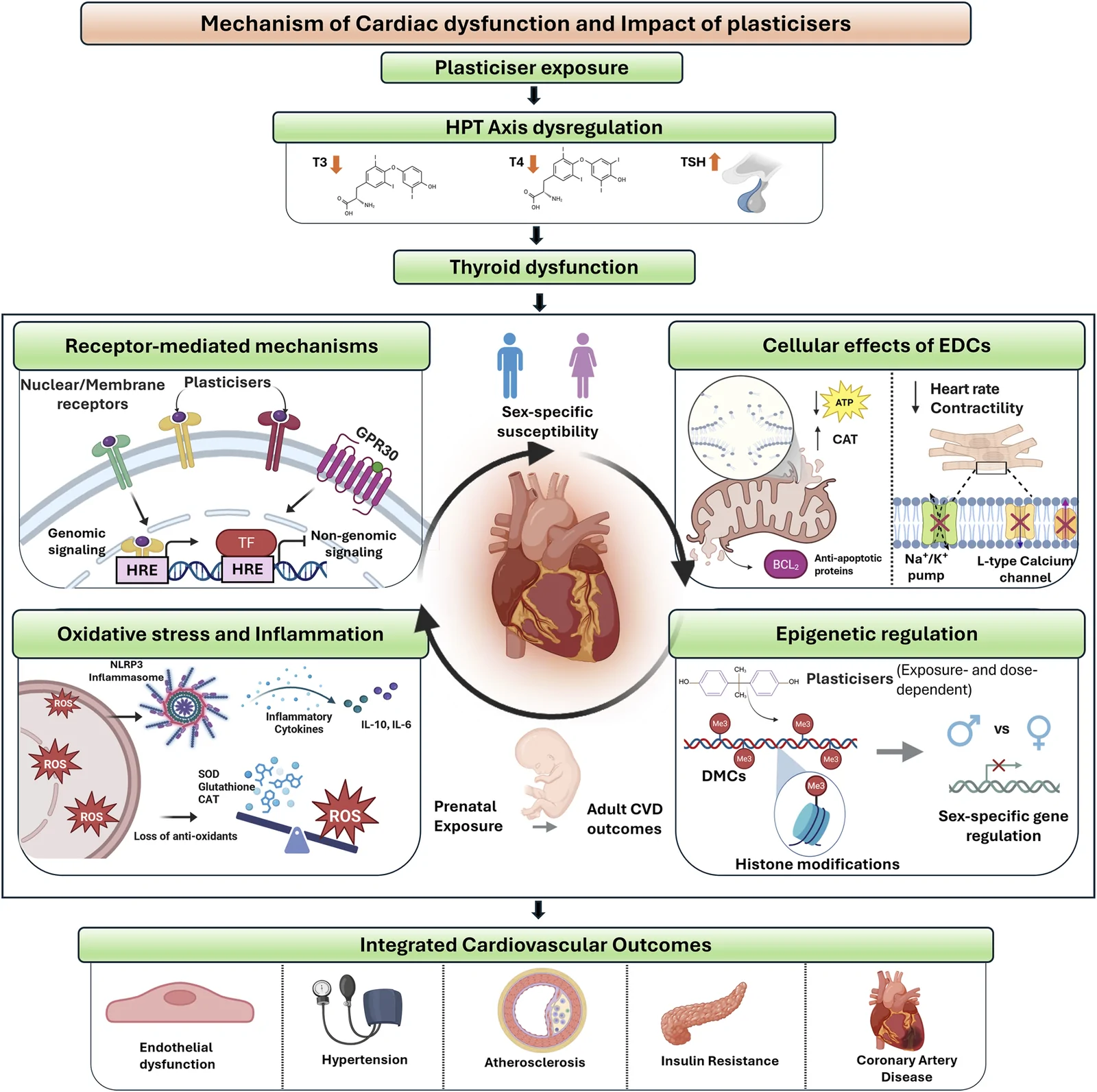
Mechanisms of cardiac dysfunction and cardiovascular toxicity induced by Plasticiser exposure. Plasticisers contribute to cardiovascular dysfunction through multiple interconnected molecular and cellular mechanisms involving receptor-mediated signalling, oxidative stress, mitochondrial dysfunction, ion-channel dysregulation, inflammation and epigenetic modifications. Receptor-mediated pathways involve interactions with nuclear receptors, including ERs, ARs and PPARs, as well as membrane-associated receptors such as GPR30. These interactions activate both genomic and non-genomic signalling pathways, leading to altered transcription factor (TF) activity and dysregulated expression of hormone-responsive elements (HREs). At the cellular level, EDCs induce mitochondrial dysfunction, oxidative damage, impaired ATP production, altered antioxidant defence systems including catalase (CAT) and dysregulation of anti-apoptotic proteins such as BCL2. Plasticiser exposure also disrupts ion transport mechanisms, including Na+/K+ pumps and L-type calcium channels, thereby impairing cardiac excitability, contractility and heart rate regulation. Oxidative stress and inflammatory signalling are characterised by excessive ROS generation, depletion of endogenous antioxidants such as SOD, glutathione and CAT, activation of the NLRP3 inflammasome and increased production of inflammatory cytokines, including IL-6 and IL-10. In addition, Plasticisers mediate epigenetic alterations through histone modifications and DMCs, contributing to sex-specific gene regulation and susceptibility to CVD. Prenatal and chronic exposure to Plasticisers may therefore increase the risk of long-term cardiovascular abnormalities and adult cardiac disease outcomes through integrated endocrine, inflammatory and epigenetic pathways.

Circulating plasticisers are associated with the structural properties of atherosclerotic plaques, suggesting their role in vascular remodelling and cardiovascular risk (Lind and Lind, 2011; Melzer et al., 2010). These effects are mediated by receptors, including nuclear, cytoplasmic and membrane-bound receptors, which regulate cellular response *via* ligand-specific activation, nuclear translocation and interact with the hormone response elements to control gene transcription (Combarnous and Nguyen, 2019). Key receptors affected include ERα, ERβ, AR, PXR,PPARα, PPARγ, thyroid TRα, TRβ, retinoid X receptors (RXR), constitutive androstane receptor (CAR), progesterone, glucocorticoid, mineralocorticoid, retinoic acid receptors farnesoid X receptor (FXR) and liver X receptors (LXR) (Combarnous and Nguyen, 2019). Beyond nuclear receptor interaction, they also affect receptor-mediated pathways, such as G protein-cAMP, phospholipase C (PLC)/protein kinase C (PKC), tyrosine kinase signalling and calcium flux (Combarnous and Nguyen, 2022). Emerging evidence further indicates that plasticisers influence alternative signalling pathways like G protein-coupled receptors (GPCR), e. g., GPER/GPR30 and estrogen-related receptors (Toporova and Balaguer, 2020; Amir et al., 2021) (Figure 2).

### 5.2 Mechanism of plasticisers in oxidative stress and inflammation

Oxidative stress is a fundamental mechanism underlying plasticiser-induced cardiotoxicity and results from an imbalance between the generation of reactive oxygen species (ROS) and cellular antioxidant defence system, leading to lipid peroxidation, protein oxidation, DNA damage and impaired cellular function (de Santana et al., 2026; Liu et al., 2022). Plasticiser exposure activates multiple redox-sensitive signalling pathways, including Nuclear Factor kappa-light-chain-enhancer of activated B cells (NF-κB), c-Jun N-terminal kinase, Activator Protein-1 (AP-1) and PI3K/Akt (Lee et al., 2021; D’Amico et al., 2022) thereby promoting inflammatory responses, endothelial dysfunction and metabolic disturbances (Ghassabian and Trasande, 2018). In addition to enhancing oxidative stress, plasticisers activate the NLR family pyrin domain-containing 3 (NLRP3) inflammasome, resulting in increased production of pro-inflammatory cytokines and tissue injury. Collectively, these molecular events establish oxidative stress and chronic inflammation as central mechanisms linking plasticiser exposure to cardiovascular dysfunction (Mariana and Cairrao, 2020).

*In vivo* studies support these mechanistic observations (Brassea-Pérez et al., 2022). For example, Zebrafish embryo studies showed concentration-dependent increases in ROS generation, activation of redox-sensitive transcription factors (de Santana et al., 2026), elevated nitric oxide production and upregulation of immune-related genes, including pro-inflammatory cytokines such as Interleukin-1 beta (IL-1β), Tumor necrosis factor-alpha and Interferon-gamma (IFN-*γ*) (Xu et al., 2013). Similarly, rodent studies demonstrated that BPA exposure induces cardiotoxicity characterised by increased oxidative stress, reduced nitric oxide bioavailability (Aboul Ezz et al., 2015) and activation of inflammatory signalling pathways, supporting the role of oxidative damage in BPA-mediated cardiovascular injury (Kovacic, 2010).

Cell-based studies extend these findings. DEHP, BPA and DINCH increased intracellular ROS production, activated NF-κB signalling, disrupted glutathione homeostasis and promoted inflammatory responses in cardiomyocytes and macrophages (Íñigo-Catalina et al., 2024; Duan et al., 2017). BPA also increase malondialdehyde (MDA) levels, depleted antioxidant enzymes (glutathione (GSH), superoxide dismutase (SOD) and catalase (CAT)) and altered glucose and lipid metabolism (Duan et al., 2017). Likewise, DINCH and its metabolite activated NF-κB signalling even at relatively low concentrations, indicating that replacement plasticisers may also possess pro-inflammatory properties (Schaffert et al., 2021). Human studies provide complementary evidence. Higher urinary concentrations of phthalate metabolites and BPA were associated with elevated inflammatory biomarkers (C-reactive protein (CRP), High-Sensitivity C-reactive protein (hsCRP), and interleukin-6 (IL-6)) together with elevated oxidative stress markers, including MDA, thiobarbituric acid reactive substances, lipid peroxidation markers (F2- isoprostanes), DNA/RNA oxidation markers (8-oxoDG, eight-oxoGuo), redox indicators (GSSG/GSH ratio) (Neier et al., 2015). These findings support experimental evidence that oxidative stress and inflammation are key mediators of plasticiser-induced cardiovascular toxicity.

### 5.3 Cellular effects of plasticisers

#### 5.3.1 Mitochondrial dysfunction

Experimental studies consistently demonstrate that conventional plasticisers such as DEHP and BPA impair mitochondrial function by disrupting oxidative phosphorylation, reducing ATP production, promoting apoptosis and altering lipid metabolism (Jin et al., 2019), thereby contributing to cardiomyocyte dysfunction (Jin et al., 2019). At the molecular level, plasticiser exposure has also been associated with mitochondrial DNA dysregulation (Fan et al., 2023), activation of autophagy (Zhou et al., 2020; Nayak et al., 2022) and apoptotic signalling (Zhou et al., 2020; Nayak et al., 2022), increased expression of pro-apoptotic mediators (e.g., BAX, BAK, cytochrome c and caspase-3) and suppression of anti-apoptotic proteins such as Bcl-2 (Wang et al., 2021).

*In vitro* studies show that DEHP impairs mitochondrial dysfunction by disrupting calcium homeostasis, activating ERK/NF-κB signalling and PPARγ-dependent metabolic dysregulation, ultimately promoting cardiomyocyte apoptosis and impaired cellular function (Giuliani et al., 2020). *In vivo* studies further show that DEHP exposure is associated with insulin resistance, dyslipidaemia and adverse cardiometabolic outcomes, whereas BPA elevates cardiac injury biomarkers, including creatine kinase-myocardial band and lactate dehydrogenase, while disrupting lipid metabolism. Collectively, these findings support mitochondrial dysfunction as a key mechanism underlying plasticiser-induced cardiotoxicity (Ghosh et al., 2010; Yang et al., 2024; Vanani et al., 2020).

#### 5.3.2 Ion channel disruption

Proper regulation of cardiac ion channels is essential for maintaining electrical conduction, excitation-contraction coupling, intracellular calcium homeostasis and normal myocardial contractility (Soriano et al., 2016; Rodrigues et al., 2020). Plasticisers disrupt these tightly regulated processes by altering the expression and function of multiple ion channels and calcium-handling proteins, thereby impairing cardiac electrophysiology and increasing susceptibility to arrhythmias and contractile dysfunction (Asano et al., 2010; Chen et al., 2025). These effects primarily involve voltage-gated sodium and calcium channels, transient receptor potential channels, potassium channels and key calcium transport systems that collectively regulate intracellular calcium dynamics and cardiac electrical activity (Rodrigues et al., 2020; Chen et al., 2025; Posnack et al., 2015; Mariana et al., 2018; Deutschmann et al., 2013).

*In vitro* studies have demonstrated that BPA directly inhibits voltage-gated sodium channels as well as T-type and L-type calcium channels (Mariana et al., 2018), resulting in impaired electrical activity and reduced cardiomyocyte contractility (Lee et al., 2025). BPA also alters transient receptor potential canonical 3 (TRPC3) expression (Chen et al., 2025), disrupts intracellular calcium homeostasis (Posnack et al., 2015) and rapidly activates large-conductance calcium-activated potassium (BK) channels in coronary smooth muscle cells. Similarly, DEHP impairs calcium handling by activating transient receptor potential vanilloid 1 (TRPV1) channels and the reverse-mode sodium/calcium exchanger (NCX), with additional involvement of PKC, potassium channels and sarco/endoplasmic reticulum calcium ATPase (SERCA), ultimately leading to negative chronotropic and inotropic effects and reduced cardiomyocyte contractility (Rodrigues et al., 2020).

*In vivo* studies further support these observations by demonstrating that plasticiser-induced disruption of calcium signalling contributes to impaired cardiac function, altered vascular smooth muscle responsiveness and increased susceptibility to cardiac electrical abnormalities and arrhythmias (Deutschmann et al., 2013; Asano et al., 2010). These findings indicate that disturbances in ion-channel function represent an important mechanism underlying plasticiser-induced cardiovascular toxicity. Comparative experimental studies further suggest that the cardiotoxic potential varies among different bisphenol analogues. While BPA consistently produces pronounced inhibitory effects on cardiac ion-channel activity, BPS exerts comparatively weaker effects on cardiac electrophysiology and ion-channel function, indicating a relatively lower cardiotoxic potential (Prudencio et al., 2021).

#### 5.3.3 Epigenetic modifications

Epigenetic modifications are heritable changes in gene expression that occur without alterations in the underlying DNA sequence and represent an important mechanism through which plasticisers may exert long-term cardiovascular effects (Handy et al., 2011). The principal epigenetic mechanisms include DNA methylation, histone modifications and non-coding RNAs mediated regulation, all of which influence chromatin architecture, transcriptional activity and cellular phenotype (Handy et al., 2011). By altering these regulatory processes, plasticisers can disrupt genes involved in lipid metabolism, inflammation, oxidative stress, cardiac development and vascular homeostasis, thereby contributing to the development and progression of cardiometabolic diseases (Handy et al., 2011; Helsley and Zhou, 2017). Human studies have identified significant associations between plasticiser exposure and epigenetic alterations. DNA methylation profiling has revealed sex-specific methylation patterns associated with urinary phthalate metabolites, with females generally exhibiting higher exposure levels, whereas males showed a greater number of differential methylated patterns of Cytosine-phosphate-Guanine (CpG) sites (Wu et al., 2025). These methylated genes were involved in immune regulation, ion channel binding, protein and nucleotide binding and membrane signalling and protein and nucleotide binding (Wu et al., 2025).

Similarly, prenatal BPA exposure has been associated with differentially methylated CpG sites (DMCs) in males (Khodasevich et al., 2024) linked to metabolic traits, including glycated haemoglobin (HbA1c) and blood pressure, suggesting that epigenetic biomarkers may provide mechanistic insight into plasticiser-associated cardiometabolic risk (Lu et al., 2020). *In vivo* studies further demonstrate that BPA induces epigenetic reprogramming in experimental animal models. Mouse studies have shown that BPA alters DNA methyltransferase expression, resulting in cardiac remodelling, dysregulated lipid metabolism and increased expression of lipogenic genes, including sterol regulatory element-binding transcription factor 1 (SREBF1) and sterol regulatory element-binding transcription factor 2 (SREBF2), thereby contributing to metabolic dysfunction (Patel et al., 2013; Ke et al., 2016). Similarly, zebrafish models have demonstrated that BPA exposure increases histone acetylation, enhances the expression of cardiac developmental genes such as Heart and Neural Crest Derivatives Expressed and induces cardiac malformation (Bouwmeester et al., 2016). Importantly, treatment with epigallocatechin gallate reversed these molecular and structural abnormalities, highlighting the potential reversibility of BPA-induced epigenetic changes (Lombó et al., 2019). Collectively, these findings indicate that epigenetic dysregulation represents an important mechanism linking plasticiser exposure to long-term cardiovascular and metabolic dysfunction.

## 6 Microfluidic platforms for detection of cardiac disorders

Conventional fluorescence-based cardiotoxicity assays, including high-throughput screening platforms such as FLIPR Penta and EarlyTox, enable rapid assessment of membrane potential and intracellular calcium dynamics but are limited by static culture conditions and their inability to reproduce physiological tissue interactions (Burnett et al., 2021; Khurana et al., 2021). In contrast, microfluidic heart-on-a-chip systems recreate dynamic flow and multicellular cardiac microenvironments, allowing real-time monitoring of contractility, electrophysiology and drug responses under physiologically relevant conditions (Agarwal et al., 2013; Munawar and Turnbull, 2021). These platforms provide physiologically relevant models for investigating the mechanisms of plasticiser-induced toxicity (Liu X. et al., 2024). Integration of microelectrodes and optical sensors enables label-free, continuous detection. Advanced 3D heart-on-chip systems support cardiac bodies or spheroids, thereby improving physiological relevance. Multi-tissue micro-physiological systems further integrate cardiac modules with liver or vascular compartments using multilayer PDMS bonding or hybrid PDMS-glass fabrication, thereby enabling detection of systemic toxicity, including plasticiser-induced cardiac abnormalities (Munawar and Turnbull, 2021) (Table 2).

**TABLE 2:** This table summarizes microfluidic and organ-on-chip platforms for the detection of thyroid and cardiac dysfunction.

Application	Microfluidic platform	Principle/Biological model	Key readouts	Advantages over conventional methods	Ref.
Cardiac dysfunction	Heart-on-a-chip	Human iPSC-derived cardiomyocytes cultured under physiological flow and mechanical stimulation	Beating frequency, contractility, calcium flux, electrophysiology	Real-time monitoring; mimics native cardiac microenvironment	102
Cardiac dysfunction	Cardiac spheroid/microtissue chip	3D cardiac spheroids containing cardiomyocytes, fibroblasts and endothelial cells	Tissue viability, contractile force, drug-induced toxicity	Improved physiological relevance compared with 2D cultures	105
Cardiac dysfunction	Microelectrode-integrated cardiac chip	Embedded microelectrodes enable continuous electrical recording	Field potential duration, arrhythmias, conduction velocity	Label-free, non-invasive, continuous monitoring	126
Cardiac dysfunction	Multi-organ heart-liver chip	Interconnected cardiac and hepatic compartments	Drug metabolism-dependent cardiotoxicity	Evaluates systemic toxicity and metabolite effects	127
Thyroid dysfunction	Thyroid-on-a-chip	Perfused thyroid follicular cells or thyroid tissue maintained under microfluidic flow	T3, T4 secretion, TSH response, hormone dynamics	Continuous assessment of thyroid function	114
Thyroid dysfunction	Thyroid tissue slices microfluidic platform	*Ex vivo* thyroid tissue maintained under controlled perfusion	Follicular integrity, hormone synthesis, cell viability	Preserves native tissue architecture and function	109
Thyroid dysfunction	Endocrine disruption screening chip	Dynamic exposure of thyroid cells to endocrine-disrupting chemicals	Hormone production, oxidative stress, inflammatory markers	Sensitive detection of low-dose endocrine disruptors	126
Thyroid-cardiac dysfunction	Integrated thyroid-heart-on-chip	Coupled thyroid and cardiac tissues connected by microchannels	Thyroid hormone secretion, cardiac contractility, rhythm changes	Direct assessment of thyroid-cardiac crosstalk	129
Plasticiser toxicity	Multi-organ microphysiological system (MPS)	Combined thyroid, liver, vascular and cardiac tissues exposed to plasticisers	Hormonal changes, cardiac biomarkers, oxidative stress, inflammation	Models chronic low-dose exposure and systemic responses	110
Biomarker detection	Biosensor-integrated microfluidic chip	On-chip electrochemical or optical biosensors	T3, T4, TSH, troponin, BNP, cytokines, ROS biomarkers	Rapid, multiplexed, low-volume, point-of-care analysis	130

### 6.1 Microfluidic devices for thyroid disorder detection

Compared with cardiac systems, thyroid-on-chip platforms are still emerging but show considerable promise for studying thyroid dysfunction. Conventional thyroid diagnostics primarily rely on endpoint measurements of T3, T4 and TSH using Enzyme-Linked Immunosorbent Assay (ELISA) or chemiluminescence assays, which cannot capture dynamic hormone secretion (Shurbaji et al., 2023). In contrast, microfluidic thyroid-on-chip systems enable continuous perfusion and real-time monitoring while preserving thyroid tissue function and physiological hormone production (Perestrelo et al., 2015; Riley et al., 2019). These platforms facilitate the evaluation of thyroid dysfunction induced by radiation, drugs and endocrine-disrupting chemicals, providing more physiologically relevant and sensitive assessment than conventional *in vitro* assays (Riley et al., 2019).

### 6.2 Organ-on-chip and multi-organ systems for thyroid-cardiac axis detection

Integrated multi-tissue organ-on-chip and micro physiological systems (MPS) allow the combination of multiple tissues on a single microfluidic chip (Donoghue et al., 2021). Integrated organ-on-chip platforms combine thyroid and cardiac tissues within a single microphysiological system, enabling investigation of thyroid-cardiac crosstalk under physiologically relevant conditions. These systems enable simultaneous assessment of endocrine disruption and cardiovascular responses following plasticiser exposure (Filion et al., 2025). Alterations in thyroid hormone secretion can therefore be directly correlated with changes in cardiac contractility, electrophysiology and rhythm within the same experimental platform (Yamakawa et al., 2021). This integrated approach provides mechanistic insights that are difficult to obtain using conventional *in vitro* models.

### 6.3 Microfluidic detection of plasticiser-mediated abnormalities

Nowadays, the detection of EDC-induced abnormalities is increasingly performed on microfluidic platforms (Wu et al., 2023). These systems will be useful for studies of chronic low-dose exposures relevant to real-world effects of EDCs (Aralekallu et al., 2023). Multiplexed, real-time monitoring of biomarkers is achieved by the integration of on-chip biosensors, enabling organ-on-chip platforms to better capture the dynamic biological responses associated with real-world environmental and chemical exposures. Continuous assessment of thyroid hormones (T3, T4 and TSH), cardiac biomarkers (troponins and B-type natriuretic peptide (BNP) and markers of oxidative stress and inflammatory cytokines provides a comprehensive assessment of exposure-induced organ dysfunction and toxicity (Omran et al., 2022). These capabilities make organ-on-chip systems particularly useful for assessing chronic, low-dose and complex exposure scenarios that are more physiologically relevant to human conditions than traditional *in vitro* models (Wu et al., 2023; Cai et al., 2026).

### 6.4 Future perspectives and translational potential of microfluidic platforms

Microfluidic devices offer several advantages over conventional diagnostic systems, including continuous real-time monitoring, low sample requirements, high-throughput screening, reduced analysis time and low-cost operation (Omran et al., 2022; Cai et al., 2026; Pal et al., 2026; Varshini et al., 2025; Spînu et al., 2021; Carrington et al., 2022; Rashid et al., 2024; Mladěnka et al., 2018; Fernández-Alvarez et al., 2025; Fröhlich and Wahl, 2017; Chaudhary and Bano, 2013; Puthiyachirakal et al., 2025; Wang et al., 2025; Ferrari et al., 2021; Ramesh et al., 2026; Pingitore et al., 2025; Liu Z. et al., 2024). They also provide physiologically relevant environments with controlled flow dynamics and enhanced cell-cell interactions, making them well suited for studying EDC-mediated cardiovascular disorders caused by hypothyroidism. Importantly, these real-time platforms enable multiplex detection and organ crosstalk modelling, both of which are essential for understanding the complex interactions between thyroid and cardiovascular dysfunction.

Recent advances in transparent glass-based surface microfluidics further enhance their biomedical potential. Laser-textured glass microfluidic platforms enable rapid, affordable and pump-free diagnostics, offering high optical clarity, biocompatibility and durable wettability patterning (Cai et al., 2026). These systems support real-time imaging, antibody functionalization, multiplex analysis and sensitive biomarker detection, delivering results quickly with minimal infrastructure and low operational costs. Future work should prioritise improving material compatibility, sensor integration, automation and device standardisation to enable clinical translation on a mobile health platform with a view to creating an efficient triage capable of categorizing patients in the order of disease severity (Pal et al., 2026). Incorporating patient-derived cells, iPSC-based models, organ-on-chip systems and AI-assisted biosensing technologies will form the rationale for developing multimodal-multifusion technologies with accuracy and sensitivity exceeding 95%, thereby facilitating their large-scale deployment in both clinical and community settings and enunciating the concepts of personalized medicine and precision public health and further enhancing personalized detection and risk assessment (Pal et al., 2026).

## 7 Conclusion and future prospects

Collectively, current evidence indicates that plasticisers disrupt the thyroid-cardiac axis through multiple interconnected endocrine and molecular mechanisms, thereby increasing the risk of thyroid dysfunction and CVD. By disrupting the thyroid-cardiac axis, plasticisers promote hypertension, atherosclerosis, arrhythmias, metabolic imbalance and cardiac dysfunction. Importantly, many alternative “phthalate-free” plasticisers may also exhibit similar toxicological effects, raising concerns about regrettable substitution. Overall, chronic low-dose and mixture exposure to plasticisers is a significant environmental risk factor for thyroid-cardiac dysfunction. Future research should prioritize long-term longitudinal human studies and realistic mixture-toxicity models to better understand the cumulative effects of plasticiser exposure. Advanced multi-omics approaches, exosomal microRNAs (Mitra et al., 2024) and epigenetic biomarkers may improve early diagnosis and mechanistic understanding of plasticiser-induced thyroid and cardiovascular disorders (Varshini et al., 2025). Despite growing evidence linking plasticiser exposure to thyroid dysfunction and CVD, several important knowledge gaps remain. Current evidence is limited by the scarcity of longitudinal human studies, insufficient evaluation of subclinical hypothyroidism, inadequate assessment of combined EDC exposures that may produce additive or synergistic effects and the limited validation of emerging biomarkers such as exosomal microRNAs. Future research should prioritise well-designed longitudinal cohort studies, realistic mixture-toxicity models, and integrated multi-omics approaches, including transcriptomics, proteomics, metabolomics, and epigenomics, to improve mechanistic understanding and biomarker discovery. Particular emphasis should also be placed on vulnerable populations, including pregnant women, infants, and occupationally exposed individuals. Addressing these gaps will facilitate the development of targeted preventive strategies, early diagnostic biomarkers and effective therapeutic interventions for plasticiser-induced thyroid-cardiac dysfunction. Emerging microfluidic and organ-on-chip technologies also provide promising platforms for real-time assessment of thyroid-cardiac toxicity. Moreover, stronger regulatory policies and safety evaluations of replacement plasticisers are urgently needed to minimize long-term public health risks. Collectively, the available evidence indicates that plasticiser-induced cardiovascular abnormalities should not be considered isolated toxicological outcomes but rather manifestations of systemic endocrine disruption, particularly involving the thyroid-cardiac axis. Integrating thyroid and cardiovascular endpoints may therefore improve mechanistic understanding, risk assessment and the development of preventive strategies against plasticiser-induced cardiometabolic disease.
